# Case conferences for infective endocarditis: A quality improvement initiative

**DOI:** 10.1371/journal.pone.0205528

**Published:** 2018-10-11

**Authors:** Charlie Tan, Mark S. Hansen, Gideon Cohen, Karl Boyle, Alvin Yang, Asgar Rishu, Ruxandra Pinto, Neill K. J. Adhikari, Nick Daneman

**Affiliations:** 1 Department of Medicine, London Health Sciences Centre, London, Ontario, Canada; 2 Division of Cardiology, Department of Medicine, Sunnybrook Health Sciences Centre, University of Toronto, Toronto, Ontario, Canada; 3 Division of Cardiac Surgery, Department of Surgery, Sunnybrook Health Sciences Centre, University of Toronto, Toronto, Ontario, Canada; 4 Division of Neurology, Beaumont Hospital, Dublin, Ireland; 5 Department of Medicine, University of Toronto, Toronto, Ontario, Canada; 6 Critical Care Research Unit, Sunnybrook Health Sciences Centre, Toronto, Ontario, Canada; 7 Department of Critical Care Medicine, Sunnybrook Health Sciences Centre, Toronto, Ontario, Canada; 8 Interdepartmental Division of Critical Care, University of Toronto, Toronto, Ontario, Canada; 9 Sunnybrook Research Institute, Toronto, Ontario, Canada; 10 Division of Infectious Diseases, Department of Medicine, Sunnybrook Health Sciences Centre, University of Toronto, Toronto, Ontario, Canada; 11 Institute for Clinical Evaluative Sciences, Toronto, Ontario, Canada; University of Bern, University Hospital Bern, SWITZERLAND

## Abstract

**Background:**

A multidisciplinary approach has been recommended for the management of patients with infective endocarditis. We evaluated the impact of multidisciplinary case conferences on morbidity, mortality, and quality of care for these patients.

**Methods:**

We conducted a quasi-experimental study of consecutive patients admitted for infective endocarditis before (2013/10/1–2015/10/12, n = 97) and after (2015/10/13–2017/11/30, n = 80) implementation of case conferences to discuss medical and surgical management. These occurred as face-to-face discussions or electronically (for non-complex patients), and included physicians from cardiac surgery, cardiology, critical care, infectious diseases and neurology. We assessed process-of-care and clinical outcomes, with the primary outcome being complications up to 90 days after hospital discharge.

**Results:**

A case conference was held for 80/80 (100%) of patients in the post-intervention group. After the intervention, more patients received inpatient cardiology assessment (81.3% [post-intervention] vs. 63.9% [pre-intervention], p = 0.01), and more patients with definite infective endocarditis underwent cardiac surgery treatment (44.6% vs. 21.7%, p = 0.007). All pre-intervention and post-intervention patients received guideline-concordant antimicrobial therapy. There was no difference in rates of complications (40.0% vs. 51.5%, p = 0.13) or mortality up to 90 days after hospital discharge (26.3% vs. 17.5%, p = 0.20). In multivariable analyses, the intervention was not associated with differences in mortality (odds ratio 1.87, 95% confidence interval 0.88–3.99) or a composite measure of complications and mortality (odds ratio 0.86, 95% confidence interval 0.46–1.58).

**Conclusion:**

We successfully implemented a standardized multidisciplinary case conference protocol for patients with infective endocarditis. This intervention had no detectable effect on complications or mortality.

## Introduction

Infective endocarditis is generally fatal if untreated. Management requires intensive medical and surgical intervention [1, 2], and morbidity and mortality remain high despite advances in diagnosis and treatment. Mortality in hospital and up to six months following hospital discharge ranges from 15 to 30% [3–13]. Complications are common, diverse and often severe, including heart failure, embolic events and neurological sequelae [2, 14, 15]. Treatment decisions are made difficult by variability in patient characteristics, comorbidities, extent of endocardial involvement, hemodynamics and microbiologic etiology. Furthermore, the coordination of multiple services is required, with 40 to 50% undergoing cardiac surgery [7, 16–18]. Fragmentation of care may lead to delays in diagnosis, inadequate antimicrobial therapy, and inappropriate indications and timing for surgery.

Recent guidelines by the European Society of Cardiology, American College of Cardiology and American Heart Association have recommended a collaborative approach to the management of infective endocarditis [1, 2], with involvement of a multidisciplinary team including cardiac surgeons, cardiologists, infectious diseases physicians, neurologists and other specialists to optimize decision-making. Three European centres that implemented a team-based model for managing infective endocarditis demonstrated significant reductions in mortality [19–21]. However, such approaches have not been evaluated in a North American context.

We established a multidisciplinary working group that participated in individualized case conferences on patients with infective endocarditis, advising on diagnosis and management according to best available evidence and clinical judgment. The objective of this study was to evaluate the impacts of this team-based approach on quality of care process measures and clinical outcome measures, including complications and mortality.

## Methods

### Study design

This study was a quasi-experimental before-after study conducted at Sunnybrook Health Sciences Centre (SHSC), a tertiary acute care teaching hospital in Toronto, Ontario, Canada. It was divided into two periods: pre-intervention, prior to implementation of the case conference protocol (October 1 2013–October 12 2015), and post-intervention, after implementation of the protocol (October 13 2015–November 30 2017). The study duration was selected based on resources available for data collection in the pre-intervention period, and the desire to match that 2-year period with a similar duration post-intervention. We anticipated approximately one admission of infective endocarditis per week based on prior experience at our centre.

### Case conference protocol

We established a working group consisting of a member from each of the cardiac surgery, cardiology, critical care, infectious diseases and neurology services. A standardized protocol was established to facilitate case conference implementation (S1 Fig), and local treatment guidelines were developed based on the American Heart Association guidelines for infective endocarditis [1]. The case conference protocol and treatment guidelines were circulated across the involved services for input. Beginning October 13 2015, the working group was notified following the identification of a patient with infective endocarditis. Notification occurred by the admitting service (cardiac surgery, cardiology, critical care or general internal medicine) or by the infectious diseases consultant. This notification initiated an electronic discussion in all cases. If the patient was deemed complex (e.g., critical care admission, embolic or neurologic complications, heart failure, concerning echocardiographic findings), a face-to-face case conference was then organized. If the patient was deemed non-complex, discussion continued electronically. Options for antimicrobial and surgical management were considered, and a consensus recommendation was communicated to the treating team and entered into the patient’s electronic medical record. The working group provided recommendations, but the physicians directly caring for or consulting on the patient were ultimately responsible for final management decisions.

### Patient identification

We included all patients admitted to SHSC during the study period who met definite modified Duke criteria or probable criteria with strong clinical suspicion for infective endocarditis [22]. Only unique patients with an active diagnosis of infective endocarditis admitted for medical or surgical management were included. Patients admitted for elective valve surgery after previously completing a full course of antimicrobial therapy or with nonbacterial thrombotic endocarditis were excluded. We also excluded patients with previously expressed wishes for non-surgical management, or rare patients who were transferred from another institution with an established definitive management plan. The sample size was determined by the number of unique patients with infective endocarditis during the study period.

Patients admitted between October 1 2013 and June 30 2015 were identified retrospectively. The hospital medical records department was screened for all patients who received an International Classification of Diseases (ICD-10-CM) code for infective endocarditis (I33, I38, I39) in any of their diagnoses. We have previously validated the accuracy of these ICD-10-CM codes in identifying patients with infective endocarditis [23]. Additional cases were detected by reviewing the hospital’s registry of heart valve repair and replacement surgeries, and the microbiology database for patients with at least one positive blood culture for microorganisms that commonly cause infective endocarditis (i.e., *Staphylococcus aureus*, *Enterococcus* spp., viridans group streptococci, *Streptococcus bovis/gallolyticus*, HACEK organisms). Patients admitted between July 1 2015 and November 30 2017 (i.e., the last three months of the pre-intervention period and the full post-intervention period) were identified prospectively by notification from the cardiac surgery, cardiology, infectious diseases or general internal medicine services.

### Patient characteristics

Patient characteristics were abstracted from electronic and paper medical records. This included patient age, sex, route of admission (direct or transfer), and Duke criteria (definite or probable); comorbidities included coronary artery disease, coronary artery bypass grafting, prosthetic valve, unrepaired valve lesion, intracardiac device, past history of infective endocarditis, intravenous drug use, intravenous instrumentation (e.g., hemodialysis, central venous catheter), hypertension, diabetes mellitus, cerebrovascular disease, chronic kidney disease, liver disease, chronic obstructive pulmonary disease and malignancy. We documented clinical features at admission, including heart failure, neurologic emboli, non-neurologic emboli and mycotic aneurysm. Microbiologic etiology was determined by microorganisms isolated from blood cultures or operative specimens. We also recorded endocardial involvement on echocardiography, including valves or devices affected, maximum vegetation diameter, vegetation hypermobility, valve disruption, abscess and fistula.

### Process measures

We examined two types of process measures to assess the fidelity of the intervention. Protocol adherence measures included holding of a working group discussion (electronic or face-to-face case conference), days to face-to-face case conference from hospital admission (for patients for whom there was a face-to-face meeting), formulation of a treatment recommendation, and entry of the recommendation into the electronic medical record. Hospital care measures included assessment by cardiac surgery, cardiology and infectious diseases consultation services following admission, cardiac surgery procedure (i.e., valve repair or replacement, removal of intracardiac device), days to cardiac surgery from admission, and follow-up by cardiac surgery, cardiology and infectious diseases services after discharge. We also evaluated the appropriateness of antimicrobial choice and duration, based on microbiologic etiology and relevant patient factors (e.g., prosthetic valve, drug allergies); appropriateness was defined as concordance with American Heart Association guidelines [1].

### Patient outcomes

The primary clinical outcome was a composite measure of complications that were new or worse from admission and assessed up to 90 days after hospital discharge, defined as congestive heart failure, ischemic stroke, hemorrhagic stroke, non-neurologic emboli, mycotic aneurysm, new arrhythmia, unexpected (i.e. post-admission and not associated with cardiac surgery) or prolonged critical care admission (i.e., beyond expected duration for recovery following cardiac surgery), or renal replacement therapy. Secondary outcomes (assessed from admission up to 90 days after hospital discharge) included all-cause mortality, all-cause readmission, attributable readmission, and relapse of infective endocarditis, as well as hospital mortality and total length of hospital stay during the index admission. Outcomes following discharge were obtained from the patient’s electronic medical record, which records follow-up at the study hospital but not at other hospitals or with community-based physicians. Patients without any records post-discharge were deemed lost to follow-up. Attributable readmission was defined as being secondary to a complication of infective endocarditis (e.g., congestive heart failure from vegetation-related valvular disease, new embolic phenomena, central venous access-related blood stream infection, surgical site infection post-cardiac surgery) or the index admission (e.g., hospital-acquired pneumonia, *Clostridium difficile* colitis), and was assessed by one investigator via review of post-discharge records. Patient characteristics, process measures and outcomes were abstracted with a standardized case report form (FileMaker Pro 12.0v2, Santa Clara, CA, USA).

### Statistical analysis

We performed descriptive analyses of the pre-intervention and post-intervention groups, comparing them by demographic characteristics, comorbidities, clinical presentation at admission, microbiologic etiology and endocardial involvement. Protocol adherence measures were described for the post-intervention group. Hospital care measures and patient outcomes were compared between the pre-intervention and post-intervention groups. Discrete variables were reported as frequencies with percentages and compared using Fisher’s exact test. Continuous variables were reported as medians with interquartile ranges and compared using the Wilcoxon rank sum test. In addition, multivariable logistic regression analyses were conducted to determine the effects of the intervention on all-cause mortality up to 90 days after hospital discharge, as well as a composite measure of mortality and complications. Age, *S*. *aureus* infective endocarditis and presence of heart failure at admission were selected *a priori* as the covariates based on their expected prognostic value. We limited the number of predictor variables to four based on the number of anticipated events, targeting at least five events per variable (24).

We performed a sensitivity analysis limited to patients who met definite modified Duke criteria. We also conducted an analysis limited to patients in the post-intervention group, comparing those who had electronic discussions with those who had face-to-face case conferences.

All analyses were performed with Microsoft Excel (Microsoft Corporation, Redmond, WA, USA) and R statistical software version 3.2.2 (R Foundation for Statistical Computing, Vienna, Austria).

### Ethics

This study was approved by the Sunnybrook Health Sciences Centre Research Ethics Board, which waived the requirement for informed consent.

## Results

### Study population

During the study period, 177 patients with infective endocarditis were admitted to SHSC, with 97 patients in the pre-intervention group and 80 patients in the post-intervention group. The characteristics of the included patients are shown in Table 1. Chronic obstructive pulmonary disease was more common in the post-intervention group; no other significant differences in demographic characteristics, comorbidities or clinical presentation were observed. Viridans group streptococci were the most common microorganism identified (26.6%), followed by *S*. *aureus* (25.4%) and *Enterococcus* spp. (16.4%). Native valve involvement was identified on echocardiography in 61.0% of cases, with the mitral valve being most commonly affected (37.3%). Microbiologic etiology and valvular involvement were similar between the two periods, with greater intracardiac device-associated infective endocarditis in the post-intervention group (7.5% vs. 1.0%, p = 0.05).

**Table 1:** Patient characteristics, microbiologic etiology and endocardial involvement of pre-intervention and post-intervention groups.

Characteristic (%)	Overall (p = 177)	Pre-intervention (n = 97)	Post-intervention (n = 80)	p value
***Demographic characteristics***				
Mean age, in years (standard deviation)	65.0 (17.6)	65.4 (19.2)	64.5 (15.5)	0.74
Male	124 (70.1)	66 (68.0)	58 (72.5)	0.62
Route of admission				
Direct	109 (61.6)	61 (62.9)	48 (60.0)	0.76
Transfer	68 (38.4)	36 (37.1)	32 (40.0)	
***Duke criteria***				
Definite	125 (70.6)	69 (71.1)	56 (70.0)	0.87
Probable	52 (29.4)	28 (28.9)	24 (30.0)	
***Comorbidities***				
Coronary artery disease	43 (24.3)	26 (26.8)	17 (21.3)	0.48
Coronary artery bypass grafting	19 (10.7)	11 (11.3)	8 (10.0)	0.81
Prosthetic valve(s)	57 (32.2)	32 (33.0)	25 (31.3)	0.87
Unrepaired valve lesion	36 (20.3)	23 (23.7)	13 (16.3)	0.63
Intracardiac device	23 (13.0)	12 (12.4)	11 (13.8)	0.83
Prior infective endocarditis	22 (12.4)	12 (12.4)	10 (12.5)	1
Intravenous drug use	14 (7.9)	8 (8.2)	6 (7.5)	1
Intravenous instrumentation (hemodialysis, central venous line)	8 (4.5)	4 (4.1)	4 (5.0)	1
Hypertension	92 (52.0)	49 (50.5)	43 (53.8)	0.76
Diabetes mellitus	46 (26.0)	22 (22.7)	24 (30.0)	0.30
Cerebrovascular disease	32 (18.1)	20 (20.6)	12 (15.0)	0.43
Chronic obstructive pulmonary disease	9 (5.1)	8 (8.2)	1 (1.3)	*0*.*04*
Chronic kidney disease	34 (19.2)	17 (17.5)	17 (21.3)	0.57
Liver disease	14 (7.9)	8 (8.2)	6 (7.5)	1
Malignancy	41 (23.2)	27 (27.8)	14 (17.5)	0.11
***Clinical presentation at admission***				
Heart failure	59 (33.3)	38 (39.2)	21 (26.3)	0.08
Neurologic emboli	37 (20.9)	20 (20.6)	17 (21.3)	1
Non-neurologic emboli	46 (26.0)	21 (21.6)	25 (31.3)	0.17
Mycotic aneurysm	4 (2.3)	3 (3.1)	1 (1.3)	0.63
***Microbiologic etiology***				
*Staphylococcus aureus*	45 (25.4)	21 (21.6)	24 (30.0)	0.23
Viridans group streptococci	47 (26.6)	27 (27.8)	20 (25.0)	0.73
*Enterococcus* species	29 (16.4)	16 (16.5)	13 (16.3)	1
HACEK group species	4 (2.3)	2 (2.1)	2 (2.5)	1
Coagulase-negative staphylococci	10 (5.6)	6 (6.2)	4 (5.0)	1
Other microorganism	32 (18.1)	22 (22.7)	10 (12.5)	0.12
Culture-negative endocarditis	13 (7.3)	8 (8.2)	5 (6.3)	0.77
***Endocardial involvement***				
Valves or devices affected				
Native mitral valve	66 (37.3)	34 (35.1)	32 (40.0)	0.53
Native aortic valve	51 (28.8)	29 (29.9)	22 (27.5)	0.74
Native tricuspid valve	14 (7.9)	9 (9.3)	5 (6.3)	0.58
Native pulmonic valve	2 (1.1)	2 (2.1)	0 (0)	0.50
Prosthetic mitral valve	10 (5.6)	3 (3.1)	7 (8.8)	0.19
Prosthetic aortic valve	21 (11.9)	10 (10.3)	11 (13.8)	0.49
Other prosthetic valve, conduit or shunt	0 (0)	0 (0)	0 (0)	1
Intracardiac device	7 (4.0)	1 (1.0)	6 (7.5)	*0*.*05*
Multiple valves involved	25 (14.1)	10 (10.3)	15 (18.8)	0.13
No definite echocardiographic evidence of endocardial involvement	39 (22.0)	23 (23.7)	16 (20.0)	0.59
Mean maximum vegetation diameter, in cm (standard deviation)*	1.41 (0.83)	1.29 (0.67)	1.52 (0.96)	0.18
Vegetation hypermobility	59 (33.3)	27 (27.8)	32 (40.0)	0.11
Valve disruption or perforation	60 (33.9)	32 (33.0)	28 (35.0)	0.87
Abscess	23 (13.0)	11 (11.3)	12 (15.0)	0.51
Fistula	2 (1.1)	0 (0)	2 (2.5)	0.20

*Patients with documented vegetation diameters on echocardiography (pre-intervention: n = 50, post-intervention: n = 48)

HACEK = *Haemophilus* spp., *Aggregatibacter actinomycetemcomitans*, *Aggregatibacter aphrophilus*, *Cardiobacterium hominis*, *Eikenella corrodens*, *Kingella kingae*

### Protocol adherence process measures

A case conference was held for 80/80 (100%) of patients in the post-intervention group, with 49 (61.3%) conducted electronically and 31 (38.8%) conducted in person (Table 2). Every working group member participated in each of the electronic discussions. The service with the highest attendance at face-to-face case conferences was infectious diseases (n = 31), followed by cardiology (n = 30), critical care (n = 25), cardiac surgery (n = 22) and neurology (n = 10). The median duration from admission to face-to-face case conference was 4 days (interquartile range 2–7 days); face-to-face conferences were always (n = 31) preceded by earlier electronic discussion. A consensus recommendation was made for all patients, with medical management (57.5%) and urgent surgery (23.8%) being most common. A recommendation was entered into the electronic medical record for 68.8% of cases (Table 2). An entry was made following 29/31 (93.5%) face-to-face conferences, compared to 26/49 (53.1%) electronic discussions (p<0.001).

**Table 2:** Protocol adherence process measures in post-intervention group.

Outcome (%)	Post-intervention (n = 80)
Working group discussion	80 (100)
Electronic	49 (61.3)
Face-to-face case conference	31 (38.8)
Median time from admission to case conference, in days (interquartile range)	4 (2–7)
Recommendation made	
Emergent surgery	4 (5.0)
Urgent surgery	19 (23.8)
Elective surgery, prior to discharge	2 (2.5)
Elective surgery, after discharge	2 (2.5)
Surgery decision pending, re-assess in hospital	2 (2.5)
Surgery decision pending, re-assess after discharge	1 (1.3)
Medical management	46 (57.5)
Recommendation entered into electronic medical record	55 (68.8)

### Hospital care process measures

More patients received formal inpatient assessment by the cardiology consult service (independent of face-to-face case conferences) during the post-intervention period (81.3% [post-intervention] vs. 63.9% [pre-intervention], p = 0.01) (Table 3). There were no significant differences in rates of assessment by the other consultation services. There was a trend toward more patients in the post-intervention group undergoing surgery for valve repair, valve replacement or intracardiac device removal than in the pre-intervention group (35.0% vs. 21.6%, p = 0.06), but time to surgery was similar (Table 3). All patients in both periods received an appropriate choice and duration of antimicrobial therapy based on local treatment protocols (Table 3).

**Table 3:** Hospital care process measures of pre-intervention and post-intervention groups.

Outcome (%)	Pre-intervention (n = 97)	Post-intervention (n = 80)	p value
Assessments performed			
Cardiac surgery	57 (58.8)	39 (48.8)	0.22
Cardiology	62 (63.9)	65 (81.3)	*0*.*01*
Infectious diseases	97 (100)	79 (98.8)	0.45
Cardiac surgery performed	21 (21.6)	28 (35.0)	0.06
Median time from admission to surgery, in days (interquartile range)	8 (5–14)	6.5 (3.75–11.25)	0.43
Appropriate antimicrobial agent	97 (100)	80 (100)	1
Appropriate antimicrobial duration	97 (100)	80 (100)	1
Follow-up*			
Cardiac surgery	16 (19.3)	18 (28.1)	0.24
Cardiology	17 (20.5)	21 (32.8)	0.13
Infectious diseases	53 (63.9)	38 (59.4)	0.61

*Excluding hospital deaths (pre-intervention: n = 83, post-intervention: n = 64)

### Patient outcomes

Among our entire cohort, 82/177 (46.3%) patients experienced complications after admission to hospital and up to 90 days after hospital discharge (Table 4). There were non-significant reductions in overall complications (40.0% [post-intervention] vs. 51.5% [pre-intervention], p = 0.13), congestive heart failure (11.3% vs. 19.6%, p = 0.15) and unexpected or prolonged critical care admission (12.5% vs. 23.7%, p = 0.08). Mortality up to 90 days after hospital discharge was 21.5%, and hospital mortality was 17.5%. Hospital mortality, mortality up to 90 days after hospital discharge, length of hospital stay, all-cause re-admissions and relapses were similar between groups (Table 4). Mortality among patients who underwent cardiac surgery was 9/49 (18.4%), with no significant difference between intervention periods (6/28 [21.4%, post-intervention] vs. 3/21 [14.2%, pre-intervention], p = 0.72). After adjusting for patient age, microbiologic etiology and heart failure at admission, the case conferencing intervention was not associated with a significant difference in mortality up to 90 days after hospital discharge (odds ratio 1.87, 95% confidence interval 0.88–3.99) or a composite of mortality and new or worse complications (odds ratio 0.86, 95% confidence interval 0.46–1.58) (Table 5).

**Table 4:** Patient outcomes in pre-intervention and post-intervention groups.

Outcome	Pre-intervention (n = 97)	Post-intervention (n = 80)	p value
Complications new or worse from admission (%)			
Any complication	50 (51.5)	32 (40.0)	0.13
Congestive heart failure	19 (19.6)	9 (11.3)	0.15
Ischemic stroke	7 (7.2)	4 (5.0)	0.76
Hemorrhagic stroke	7 (7.2)	2 (2.5)	0.19
Non-neurologic emboli	7 (7.2)	4 (5)	0.76
Mycotic aneurysm	0 (0)	2 (2.5)	0.20
Arrhythmia	23 (23.7)	14 (17.5)	0.36
Unexpected or prolonged critical care admission	23 (23.7)	10 (12.5)	0.08
Intra-aortic balloon pump	0 (0)	1 (1.3)	0.45
Renal replacement therapy	6 (6.2)	11 (13.8)	0.12
Median length of hospital stay, in days (interquartile range)	14 (9–26)	13.5 (7–21.25)	0.20
Loss to follow-up	8 (8.2)	2 (2.5)	0.12
Re-admissions (%)*	24 (31.6)	17 (28.3)	0.71
Attributable re-admissions*	20 (26.3)	8 (13.3)	0.09
Relapses (%)*	3 (3.9)	1 (1.7)	0.63
Hospital mortality (%)	13 (13.4)	18 (22.5)	0.16
Mortality up to 90 days after hospital discharge (%)	17 (17.5)	21 (26.3)	0.20

*Excluding losses to follow-up and patients who died during the index hospitalization (pre-intervention: n = 76, post-intervention: n = 60)

**Table 5:** Results of multivariable logistic regression for effect of the case conferencing intervention on mortality up to 90 days after hospital discharge and a composite of mortality and development of new or worse complications.

Variable	Mortality up to 90 days after hospital discharge		Composite of mortality and new or worse complications	
	OR (95% CI)	p value	OR (95% CI)	p value
Intervention	1.87 (0.88–3.99)	0.10	0.86 (0.46–1.58)	0.63
Age	1.01 (0.99–1.04)	0.25	0.99 (0.97–1.01)	0.29
*S*. *aureus*	2.10 (0.92–4.81)	0.08	1.44 (0.71–2.93)	0.31
Heart failure	2.32 (1.06–5.08)	*0*.*03*	1.24 (0.65–2.35)	0.52

CI = confidence interval, OR = odds ratio

### Sensitivity analysis

Analyses limited to patients with definite infective endocarditis by Duke criteria yielded results similar to the main analysis (S1–S5 Tables). There were 125 cases of definite infective endocarditis, including 69 patients pre-intervention and 56 patients post-intervention. All patients in the post-intervention group received a working group discussion. The surgery rate was higher post-intervention than pre-intervention (44.6% vs. 21.7%, p = 0.007). There was a trend towards decreased overall complications in the post-intervention group (41.1% vs. 58.0%, p = 0.07), but no reduction in mortality up to 90 days after hospital discharge (26.8% vs. 15.9%, p = 0.18).

Analyses limited to the post-intervention group demonstrated that patients who underwent face-to-face case conferences were younger and more likely to have definite infective endocarditis (S6 Table). There were no significant differences in other patient characteristics, microbiologic and endocardial features, hospital care process measures and patient outcomes (S7 and S8 Tables).

## Discussion

Our study demonstrates the implementation of a multidisciplinary case conference protocol for patients with infective endocarditis at one academic centre. After implementation, a face-to-face or electronic case conference discussion was held for all patients, which yielded a consensus recommendation on antimicrobial treatment and operative versus non-operative management. Younger patients and those with definite infective endocarditis were more likely to undergo face-to-face case conference than electronic discussion. All patients before and after the intervention received infectious diseases consultation and guideline-concordant antibiotic treatment, but the intervention led to more patients receiving inpatient cardiology assessment and a trend towards more patients having cardiac surgery. There were no detectable effects on overall or individual complications or mortality. Among patients with definite infective endocarditis, the intervention was associated with an increased probability of patients having cardiac surgery, but no significant reductions in complications or mortality.

The hospital and 90-day post-hospital discharge mortality rates at our centre are similar to published data [7, 8], but these rates did not improve with the case conferencing intervention despite more patients undergoing surgery. The cardiac surgery rate post-intervention was 35.0% (44.6% in those with definite endocarditis), closer to the rate of 40–50% reported elsewhere [7, 16–18]. There are several potential explanations. Several patients undergoing cardiac surgery were in critical condition, with a guarded prognosis regardless of intervention. Among patients who underwent cardiac surgery, there was no significant difference in mortality between groups. In addition, time to surgery was not reduced in the post-intervention group; previous work has shown early surgery to be associated with reduced morbidity and mortality [24–26]. Lastly, we were underpowered to assess the intervention’s effect on mortality.

Three prior studies have evaluated multidisciplinary approaches to the management of infective endocarditis, and all found significant reductions in hospital mortality after implementation: 12.7% to 4.4% [19]; 28% to 13% [20]; and 36.1% to 16.7% [21]. Two studies developed and adhered to standardized medical and surgical protocols that were uniformly applied to all patients [19, 20]. In the other study, members of the multidisciplinary collaboration were the attending physicians directly responsible for the patients’ care [21]. These studies also showed dramatic improvements in medical management, such as greater appropriateness of antimicrobial agent and duration (61.8% [post-intervention] vs. 22.7% [pre-intervention]) [19], as well as earlier diagnosis, greater use of transesophageal echocardiography, and a microbiological alert system that accelerated initiation of appropriate antimicrobial therapy [21]. In contrast, although we followed a local treatment protocol based on guidelines from the American Heart Association, recommendations made to the admitting physician were non-binding. Furthermore, the baseline rate of infectious diseases consultation at our hospital prior to the intervention was already 100%, and the rate of appropriate antimicrobial regimens and durations was 100% as well, thereby leaving no room for improvement in medical management. Therefore, any clinical impact of our intervention would have depended on changes in surgical care, whose effects may be more modest.

Prior to the implementation of case conferences, management of patients with infective endocarditis at our centre generally involved separate services acting independently as consultants. Our quality improvement initiative achieved several objectives: it formalized coordination among these specialties and provided the admitting service with a consensus recommendation to guide treatment; it increased the cardiac surgery rate; and it facilitated assessment by a cardiologist. However, areas for improvement remain. Recommendations were made for all patients in the post-intervention group, but more than 30% were not formally documented into the electronic medical record. Although such cases likely involved verbal communication of the recommendation to the admitting physician, a standardized process for record entry should be established, especially for electronic discussions. The median time from admission to face-to-face case conference was four days, which may be inadequate in cases requiring emergent or urgent surgery. However, all face-to-face case conferences were preceded by an electronic discussion that often resulted in a preliminary consensus on management.

Our study has several limitations. Data collection was retrospective for most of the pre-intervention period, which may have led to underestimation of comorbidities and complications due to incomplete chart documentation. Outcomes and follow-up after discharge were collected from the patient’s electronic medical record, but unreported complications were considered absent, and follow-up with physicians at other hospitals and community-based physicians could not be ascertained. Although uniform in-person follow-up would have improved detection of complications, it was not feasible for the retrospective phase of our study and for patients transferred from other institutions. Given that all patients received an infectious diseases consultation and guideline-concordant antimicrobial therapy at baseline, our intervention may have had limited potential to improve outcomes. Another limitation was the discussion of non-complex patients electronically, which may have had a lesser effect compared to face-to-face case conferences. We also did not record time to first e-mail correspondence, for both electronic discussions and face-to-face case conferences. Lastly, the short duration and small sample size may have limited the study’s ability to detect differences in patient outcomes. Since the primary objective of the intervention was process improvement, and sample size was limited by unique cases of infective endocarditis at our centre, the study was underpowered for clinical outcomes. Therefore, given that patients with infective endocarditis are heterogeneous in microbiologic etiology, endocardial burden and comorbidities, a larger population followed over a longer duration in multiple centres would be helpful to establish changes in outcome arising from this form of multidisciplinary intervention.

## Conclusion

In summary, we implemented multidisciplinary case conferences for patients with infective endocarditis. Participation among the involved specialties was sustained through the 24-month post-intervention period, with a consensus recommendation made for all patients. However, our intervention was not associated with changes in patient morbidity and mortality. Our experience can be used to guide the development of local protocols for team-based models at other centres. Further prospective studies, with greater sample sizes and study durations, would be helpful to confirm the utility of a multidisciplinary approach for infective endocarditis, establish procedures to facilitate implementation, and identify new strategies to improve patient outcomes.

## Supporting information

S1 Fig Protocol for multidisciplinary case conferences for patients with infective endocarditis. (PDF)

S1 Table Patient characteristics, microbiologic etiology and endocardial involvement of pre-intervention and post-intervention groups, in subset of patients with definite infective endocarditis. (PDF)

S2 Table Protocol compliance process measures in post-intervention group, in subset of patients with definitive infective endocarditis. (PDF)

S3 Table Hospital care process measures of pre-intervention and post-intervention groups, in subset of patients with definite infective endocarditis. (PDF)

S4 Table Patient outcomes in pre-intervention and post-intervention groups, in subset of patients with definite infective endocarditis. (PDF)

S5 Table Results of multivariable logistic regression for effect of the case conferencing intervention on mortality up to 90 days after hospital discharge and a composite of mortality and development of new or worse complications, in subset of patients with definite infective endocarditis. (PDF)

S6 Table Patient characteristics, microbiologic etiology and endocardial involvement of E-mail discussion and face-to-face case conference groups in post-intervention period. (PDF)

S7 Table Hospital care process measures of E-mail discussion and face-to-face case conference groups in post-intervention period. (PDF)

S8 Table Patient outcomes in E-mail discussion and face-to-face case conference groups in post-intervention period. (PDF)
