# Supplementary material for: Case conferences for infective endocarditis: A quality improvement initiative
Source: PLoS One. 2018 Oct 11;13(10):e0205528. doi: 10.1371/journal.pone.0205528 (PMC6181397; doi:10.1371/journal.pone.0205528)
Supplement: S1 Fig — (PDF) [file pone.0205528.s001.pdf]

S1 Fig. Protocol for Multidisciplinary Case Conferences for Patients with Infective Endocarditis.

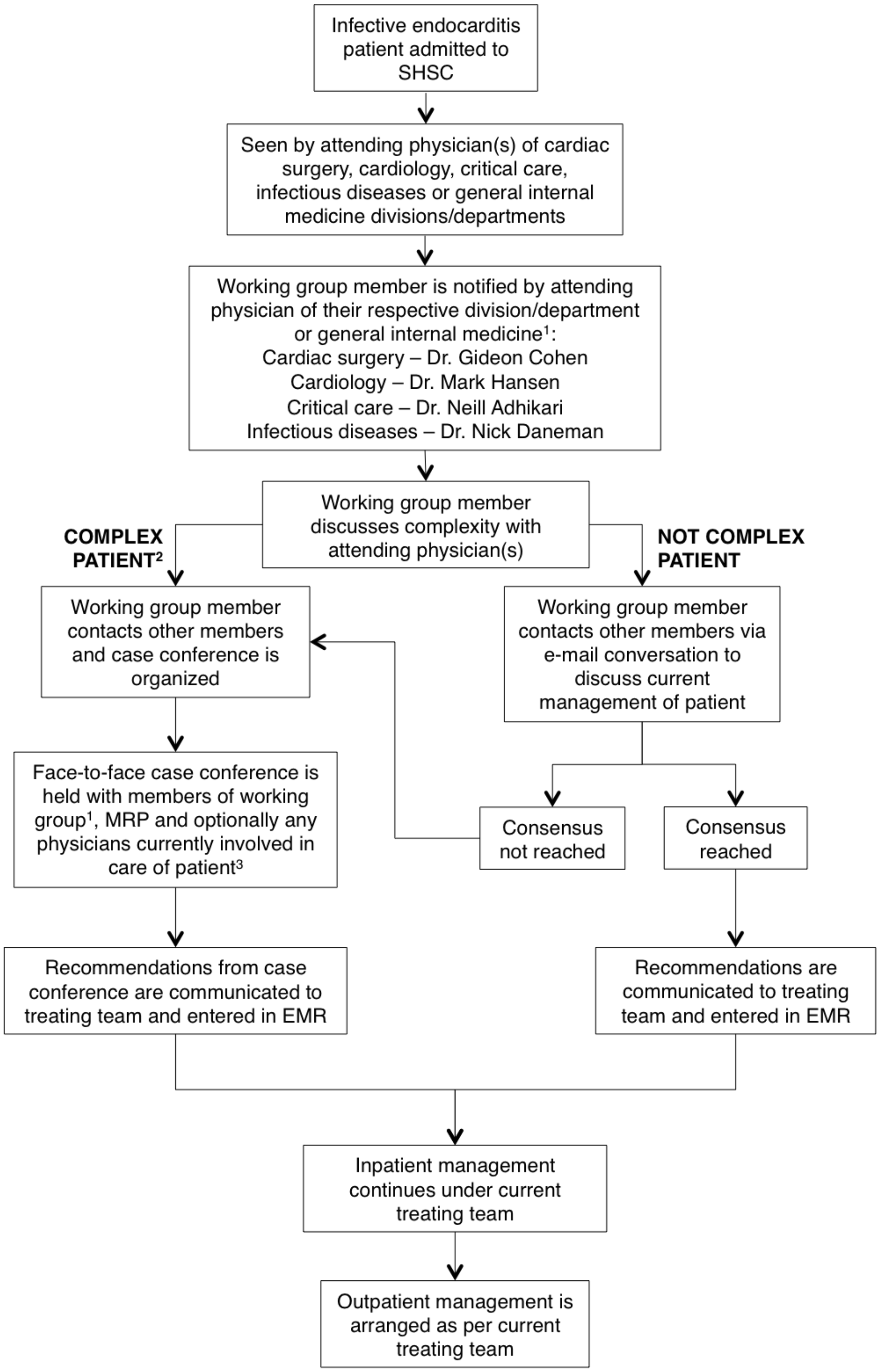

[1] Working group member may appoint a delegate if not available, most likely on-call staff for respective division/department. The delegate from the Department of Critical Care Medicine will be the CVICU attending of the week.
[2] Examples (not a comprehensive list) of complex patients include patients for whom at least one clinician or working group member thinks surgery is indicated, patients admitted to an ICU or CICU, or patients with cerebral emboli
[3] Input from neurology service may be specifically requested for cases with cerebral emboli
