# Supplementary material for: Case conferences for infective endocarditis: A quality improvement initiative
Source: PLoS One. 2018 Oct 11;13(10):e0205528. doi: 10.1371/journal.pone.0205528 (PMC6181397; doi:10.1371/journal.pone.0205528)
Supplement: S1 Table — (PDF) [file pone.0205528.s002.pdf]

S1 Table. Patient Characteristics, Microbiologic Etiology and Endocardial Involvement of Pre-Intervention and Post-Intervention Groups, in Subset of Patients with Definite Infective Endocarditis.

Characteristic (%)	Overall (n=125)	Pre-intervention (n=69)	Post-intervention (n=56)	p value
Demographic characteristics				
Mean age, in years (standard deviation)	62.1 (17.8)	62.3 (19.5)	61.9 (15.7)	0.89
Male	88 (70.4)	45 (65.2)	43 (76.8)	0.17
Route of admission				
Direct	68 (54.4)	40 (58.0)	28 (50.0)	0.47
Transfer	57 (45.6)	29 (42.0)	28 (50.0)	
Comorbidities				
Coronary artery disease	22 (17.6)	14 (20.3)	8 (14.3)	0.48
Coronary artery bypass grafting	11 (8.8)	6 (8.7)	5 (8.9)	1
Prosthetic valve(s)	32 (25.6)	17 (24.6)	15 (26.8)	0.84
Unrepaired valve lesion	28 (22.4)	19 (27.5)	9 (16.1)	0.14
Intracardiac device	12 (9.6)	5 (7.2)	7 (12.5)	0.37
Prior infective endocarditis	17 (13.6)	9 (13.0)	8 (14.3)	1
Intravenous drug use	14 (11.2)	8 (11.6)	6 (10.7)	1
Intravenous instrumentation (hemodialysis, central venous line)	6 (4.8)	4 (5.8)	2 (3.6)	0.69
Hypertension	64 (51.2)	36 (52.2)	28 (50.0)	0.86
Diabetes mellitus	32 (25.6)	15 (21.7)	17 (30.4)	0.31
Cerebrovascular disease	23 (18.4)	13 (18.8)	10 (17.9)	1
Chronic obstructive pulmonary disease	6 (4.8)	5 (7.2)	1 (1.8)	0.22
Chronic kidney disease	17 (13.6)	8 (11.6)	9 (16.1)	0.60
Liver disease	10 (8.0)	7 (10.1)	3 (5.4)	0.51
Malignancy	26 (20.8)	17 (24.6)	9 (16.1)	0.27
Clinical presentation at admission				
Heart failure	46 (36.8)	29 (42.0)	17 (30.4)	0.20
Neurologic emboli	36 (28.8)	19 (27.5)	17 (30.4)	0.84
Non-neurologic emboli	40 (32.0)	19 (27.5)	21 (37.5)	0.25
Mycotic aneurysm	4 (3.2)	3 (4.3)	1 (1.8)	0.63
Microbiologic etiology				
Staphylococcus aureus	36 (28.8)	17 (24.6)	19 (33.9)	0.32
Viridans group streptococci	30 (24.0)	19 (27.5)	11 (19.6)	0.40
Enterococcus species	21 (16.8)	12 (17.4)	9 (16.1)	1
HACEK group species	2 (1.6)	1 (1.4)	1 (1.8)	1
Coagulase-negative staphylococci	8 (6.4)	4 (5.8)	4 (7.1)	1
Other microorganism	25 (20.0)	18 (26.1)	7 (12.5)	0.07
Culture-negative endocarditis	4 (3.2)	2 (2.9)	2 (3.6)	0.81
Endocardial involvement				
Valves or devices affected				
Native mitral valve	58 (46.4)	32 (46.4)	26 (46.4)	1
Native aortic valve	44 (35.2)	24 (34.8)	20 (35.7)	1

Native tricuspid valve	14 (11.2)	9 (13.0)	5 (8.9)	0.57
Native pulmonic valve	2 (1.6)	2 (2.9)	0 (0)	0.50
Prosthetic mitral valve	9 (7.2)	3 (4.3)	6 (10.7)	0.30
Prosthetic aortic valve	19 (15.2)	9 (13.0)	10 (17.9)	0.47
Other prosthetic valve, conduit or shunt	0 (0)	0 (0)	0 (0)	1
Intracardiac device	5 (4.0)	0 (0)	5 (8.9)	*0.02*
Multiple valves involved	23 (18.4)	9 (13.0)	14 (25.0)	0.11
No definite echocardiographic evidence of endocardial involvement	4 (3.2)	3 (4.3)	1 (1.8)	0.63
Mean maximum vegetation diameter, in cm (standard deviation)*	1.43 (0.85)	1.33 (0.68)	1.54 (1.00)	0.24
Vegetation hypermobility	54 (43.2)	24 (34.8)	30 (53.6)	*0.05*
Valve disruption or perforation	55 (44.0)	29 (42.0)	26 (46.4)	0.72
Abscess	20 (16.0)	9 (13.0)	11 (19.6)	0.34
Fistula	2 (1.6)	0 (0)	2 (3.6)	0.20

*Patients with documented vegetation diameters on echocardiography (pre-intervention: n=43, post-intervention: n=44)

HACEK = *Haemophilus* spp., *Aggregatibacter actinomycetemcomitans, Aggregatibacter aphrophilus, Cardiobacterium hominis, Eikenella corrodens, Kingella kingae*
