# Supplementary material for: Case conferences for infective endocarditis: A quality improvement initiative
Source: PLoS One. 2018 Oct 11;13(10):e0205528. doi: 10.1371/journal.pone.0205528 (PMC6181397; doi:10.1371/journal.pone.0205528)
Supplement: S2 Table — (PDF) [file pone.0205528.s003.pdf]

S2 Table. Protocol Compliance Process Measures in Post-Intervention Group, in Subset of Patients with Definitive Infective Endocarditis.

Outcome (%)	Post-intervention (n=56)
Working group discussion	56 (100)
Electronic	26 (46.4)
Face-to-face case conference	30 (53.6)
Median time from admission to case conference, in days (interquartile range)	3 (2–6)
Recommendation made	
Emergent surgery	3 (5.4)
Urgent surgery	18 (32.1)
Elective surgery, prior to discharge	2 (3.6)
Elective surgery, after discharge	2 (3.6)
Surgery decision pending, re-assess in hospital	2 (3.6)
Surgery decision pending, re-assess after discharge	1 (1.8)
Medical management	25 (44.6)
Recommendation entered into electronic medical record	42 (75.0)
