# Supplementary material for: Case conferences for infective endocarditis: A quality improvement initiative
Source: PLoS One. 2018 Oct 11;13(10):e0205528. doi: 10.1371/journal.pone.0205528 (PMC6181397; doi:10.1371/journal.pone.0205528)
Supplement: S3 Table — (PDF) [file pone.0205528.s004.pdf]

S3 Table. Hospital Care Process Measures of Pre-Intervention and Post-Intervention Groups, in Subset of Patients with Definite Infective Endocarditis.

**Outcome (%)**	**Pre-intervention (n=69)**	**Post-intervention (n=56)**	**p value**
Assessments performed			
Cardiac surgery	50 (72.5)	33 (58.9)	0.13
Cardiology	48 (69.6)	51 (91.1)	*0.004*
Infectious diseases	69 (100)	55 (98.2)	0.45
Cardiac surgery performed	15 (21.7)	25 (44.6)	*0.007*
Median time from admission to surgery, in days (interquartile range)	7 (4–13.5)	7 (4–11)	0.78
Appropriate antimicrobial agent	69 (100)	56 (100)	0.78
Appropriate antimicrobial duration	69 (100)	56 (100)	1
Follow-up*			
Cardiac surgery	13 (22.8)	14 (33.3)	0.26
Cardiology	11 (19.3)	13 (31.0)	0.54
Infectious diseases	37 (64.9)	24 (57.1)	0.53

*Excluding in-hospital deaths (pre-intervention: n=57, post-intervention: n=42)
