# Supplementary material for: Case conferences for infective endocarditis: A quality improvement initiative
Source: PLoS One. 2018 Oct 11;13(10):e0205528. doi: 10.1371/journal.pone.0205528 (PMC6181397; doi:10.1371/journal.pone.0205528)
Supplement: S4 Table — (PDF) [file pone.0205528.s005.pdf]

S4 Table. Patient Outcomes in Pre-Intervention and Post-Intervention Groups, in Subset of Patients with Definite Infective Endocarditis.

Outcome	Pre-intervention (n=69)	Post-intervention (n=56)	p value
Complications new or worse from admission (%)			
Any complication	40 (58.0)	23 (41.1)	0.07
Congestive heart failure	15 (21.7)	7 (12.5)	0.24
Ischemic stroke	6 (8.7)	4 (7.1)	1
Hemorrhagic stroke	5 (7.2)	1 (1.8)	0.22
Non-neurologic emboli	7 (10.1)	4 (7.1)	0.75
Mycotic aneurysm	0 (0)	2 (3.6)	0.20
Arrhythmia	21 (30.4)	10 (17.9)	0.14
Unexpected or prolonged critical care admission	17 (24.6)	6 (10.7)	0.06
Intra-aortic balloon pump	0 (0)	1 (1.8)	0.45
Renal replacement therapy	4 (5.8)	8 (14.3)	0.13
Median length of hospital stay, in days (interquartile range)	16 (10–33)	15 (8.75–23.25)	0.29
Loss to follow-up	7 (10.1)	2 (3.6)	0.19
Re-admissions (%)*	17 (33.3)	9 (23.1)	0.35
Attributable re-admissions*	14 (27.5)	5 (12.8)	0.12
Relapses (%)*	2 (3.9)	1 (2.6)	1
Hospital mortality (%)	11 (15.9)	15 (26.8)	0.18
Mortality up to 90 days after hospital discharge (%)	13 (18.8)	16 (28.6)	0.21

*Excluding losses to follow-up and patients who died during the index hospitalization (pre-intervention: n=51, post-intervention: n=39)
