# Supplementary material for: Case conferences for infective endocarditis: A quality improvement initiative
Source: PLoS One. 2018 Oct 11;13(10):e0205528. doi: 10.1371/journal.pone.0205528 (PMC6181397; doi:10.1371/journal.pone.0205528)
Supplement: S5 Table — (PDF) [file pone.0205528.s006.pdf]

S5 Table. Results of Multivariable Logistic Regression for Effect of the Case Conferencing Intervention on Mortality Up to 90 Days After Hospital Discharge and a Composite of Mortality and Development of New or Worse Complications, in Subset of Patients with Definite Infective Endocarditis.

Variable	Mortality up to 90 days after hospital discharge		Composite of mortality and new or worse complications	
	OR (95% CI)	p value	OR (95% CI)	p value
Intervention	1.77 (0.74–4.2)	0.20	0.70 (0.34–1.44)	0.33
Age	1.02 (0.99–1.04)	0.16	0.99 (0.97–1.02)	0.60
S. aureus	1.95 (0.78–4.88)	0.16	1.18 (0.52–2.66)	0.69
Heart failure	1.40 (0.57–3.44)	0.46	0.70 (0.33–1.50)	0.36

CI = confidence interval, OR = odds ratio
