# Supplementary material for: Case conferences for infective endocarditis: A quality improvement initiative
Source: PLoS One. 2018 Oct 11;13(10):e0205528. doi: 10.1371/journal.pone.0205528 (PMC6181397; doi:10.1371/journal.pone.0205528)
Supplement: S6 Table — (PDF) [file pone.0205528.s007.pdf]

S6 Table. Patient Characteristics, Microbiologic Etiology and Endocardial Involvement of E-Mail Discussion and Face-to-Face Case Conference Groups in Post-Intervention Period.

Characteristic (%)	E-mail discussion (n=49)	Face-to-face case conference (n=31)	p value
Demographic characteristics			
Mean age, in years (standard deviation)	68.8 (13.5)	57.7 (16.1)	*0.002*
Male	13 (26.5)	9 (29.0)	0.80
Route of admission			
Direct	28 (57.1)	20 (64.5)	0.64
Transfer	21 (42.9)	11 (35.5)	
Duke criteria			
Definite	30 (61.2)	26 (83.9)	*0.04*
Probable	19 (38.8)	5 (16.1)	
Comorbidities			
Coronary artery disease	11 (22.4)	6 (19.4)	0.79
Coronary artery bypass grafting	4 (8.2)	4 (12.9)	0.70
Prosthetic valve(s)	14 (28.6)	11 (35.5)	0.62
Unrepaired valve lesion	10 (20.4)	3 (9.7)	0.35
Intracardiac device	5 (10.2)	6 (19.4)	0.32
Prior infective endocarditis	6 (12.2)	4 (12.9)	1
Intravenous drug use	2 (4.1)	4 (12.9)	0.20
Intravenous instrumentation (hemodialysis, central venous line)	2 (4.1)	2 (6.5)	0.64
Hypertension	30 (61.2)	13 (41.9)	0.11
Diabetes mellitus	16 (32.7)	8 (25.8)	0.62
Cerebrovascular disease	4 (8.2)	8 (25.8)	0.05
Chronic obstructive pulmonary disease	0 (0)	1 (3.2)	0.39
Chronic kidney disease	10 (20.4)	7 (22.6)	1
Liver disease	3 (6.1)	3 (9.7)	0.67
Malignancy	9 (18.4)	5 (16.1)	1
Clinical presentation at admission			
Heart failure	15 (30.6)	6 (19.4)	0.31
Neurologic emboli	9 (18.4)	8 (25.8)	0.58
Non-neurologic emboli	12 (24.5)	13 (41.9)	0.14
Mycotic aneurysm	1 (2.0)	0 (0)	1
Microbiologic etiology			
Staphylococcus aureus	11 (22.4)	13 (41.9)	0.08
Viridans group streptococci	15 (30.6)	5 (16.1)	0.19
Enterococcus species	8 (16.3)	5 (16.1)	1
HACEK group species	2 (4.1)	0 (0)	0.52
Coagulase-negative staphylococci	2 (4.1)	2 (6.5)	0.64
Other microorganism	6 (12.2)	4 (12.9)	1
Culture-negative endocarditis	4 (8.2)	1 (3.2)	0.64
Endocardial involvement			

Valves or devices affected			
Native mitral valve	22 (44.9)	10 (32.3)	0.35
Native aortic valve	13 (26.5)	9 (29.0)	0.80
Native tricuspid valve	2 (4.1)	3 (9.7)	0.37
Native pulmonic valve	0 (0)	0 (0)	1
Prosthetic mitral valve	5 (10.2)	2 (6.5)	0.70
Prosthetic aortic valve	6 (12.2)	5 (16.1)	0.74
Other prosthetic valve, conduit or shunt	0 (0)	0 (0)	1
Intracardiac device	1 (2.0)	5 (16.1)	*0.03*
Multiple valves involved	11 (22.4)	4 (12.9)	0.38
No definite echocardiographic evidence of endocardial involvement	12 (24.5)	4 (12.9)	0.26
Mean maximum vegetation diameter, in cm (standard deviation)*	1.40 (0.62)	1.65 (1.22)	0.37
Vegetation hypermobility	17 (34.7)	15 (48.4)	0.25
Valve disruption or perforation	17 (34.7)	11 (35.5)	1
Abscess	5 (10.2)	7 (22.6)	0.20
Fistula	1 (2.0)	1 (3.2)	1

*Patients with documented vegetation diameters on echocardiography (e-mail discussion: n=24, face-to-face case conference: n=24)
