# Supplementary material for: Case conferences for infective endocarditis: A quality improvement initiative
Source: PLoS One. 2018 Oct 11;13(10):e0205528. doi: 10.1371/journal.pone.0205528 (PMC6181397; doi:10.1371/journal.pone.0205528)
Supplement: S7 Table — (PDF) [file pone.0205528.s008.pdf]

S7 Table. Hospital Care Process Measures of E-Mail Discussion and Face-to-Face Case Conference Groups in Post-Intervention Period.

**Outcome (%)**	**E-mail discussion (n=49)**	**Face-to-face case conference (n=31)**	**p value**
Assessments performed			
Cardiac surgery	20 (40.8)	19 (61.3)	0.11
Cardiology	37 (75.5)	28 (90.3)	0.14
Infectious diseases	48 (98.0)	31 (100)	1
Cardiac surgery performed	14 (28.6)	14 (45.2)	0.15
Median time from admission to surgery, in days (interquartile range)	7 (3.5–11.0)	6.5 (4.0-11.3)	0.95
Appropriate antimicrobial agent	49 (100)	31 (100)	1
Appropriate antimicrobial duration	49 (100)	31 (100)	1
Follow-up*			
Cardiac surgery	11 (26.8)	7 (30.4)	0.78
Cardiology	14 (34.1)	7 (30.4)	1
Infectious diseases	23 (56.1)	15 (65.2)	0.60

*Excluding hospital deaths (e-mail discussion: n=41, face-to-face case conference: n=23)
