# Supplementary material for: Case conferences for infective endocarditis: A quality improvement initiative
Source: PLoS One. 2018 Oct 11;13(10):e0205528. doi: 10.1371/journal.pone.0205528 (PMC6181397; doi:10.1371/journal.pone.0205528)
Supplement: S8 Table — (PDF) [file pone.0205528.s009.pdf]

S8 Table. Patient Outcomes in E-Mail Discussion and Face-to-Face Case Conference Groups in Post-Intervention Period.

Outcome	E-mail discussion (n=49)	Face-to-face case conference (n=31)	p value
Complications new or worse from admission (%)			
Any complication	20 (40.8)	12 (38.7)	1
Congestive heart failure	5 (10.2)	4 (12.9)	0.73
Ischemic stroke	3 (6.1)	1 (3.2)	1
Hemorrhagic stroke	2 (4.1)	0 (0)	0.52
Non-neurologic emboli	2 (4.1)	2 (6.5)	0.64
Mycotic aneurysm	1 (2.0)	1 (3.2)	1
Arrhythmia	9 (18.4)	5 (16.1)	1
Unexpected or prolonged critical care admission	5 (10.2)	5 (16.1)	0.50
Intra-aortic balloon pump	1 (2.0)	0 (0)	1
Renal replacement therapy	7 (14.3)	4 (12.9)	1
Median length of hospital stay, in days (interquartile range)	14 (10.6)	13 (22.5)	0.49
Loss to follow-up	1 (2.0)	1 (3.2)	1
Re-admissions (%)*	10 (20.4)	7 (22.6)	0.79
Attributable re-admissions*	3 (6.1)	5 (16.1)	0.25
Relapses (%)*	0 (0)	1 (3.2)	0.39
Hospital mortality (%)	9 (18.4)	9 (29.0)	0.40
Mortality up to 90 days after hospital discharge (%)	11 (22.4)	10 (32.3)	0.44
